# Paper as Active Layer in Inkjet-Printed Capacitive Humidity Sensors

**DOI:** 10.3390/s17071464

**Published:** 2017-06-22

**Authors:** Cristina Gaspar, Juuso Olkkonen, Soile Passoja, Maria Smolander

**Affiliations:** Printed Functional Solutions, VTT Technical Research Centre of Finland, Espoo 02044, Finland; juuso.olkkonen@dispelix.com (J.O.); soile.passoja@elisanet.fi (S.P.); maria.smolander@vtt.fi (M.S.)

**Keywords:** inkjet printing, paper, humidity sensor, nanoparticles, silver

## Abstract

An inkjet-printed relative humidity sensor based on capacitive changes which responds to different humidity levels in the environment is presented in this work. The inkjet-printed silver interdigitated electrodes configuration on the paper substrate allowed for the fabrication of a functional proof-of-concept of the relative humidity sensor, by using the paper itself as a sensing material. The sensor sensitivity in terms of relative humidity changes was calculated to be around 2 pF/RH %. The response time against different temperature steps from 3 to 85 °C was fairly constant (about 4–5 min), and it was considered fast for the aimed application, a smart label.

## 1. Introduction

The use of paper as flexible substrate material for printed electronics has been increasing when compared to polymer-based substrates. This is due to its attractive characteristics, such as being a widely available substrate and a low-cost material. Its flexibility allows a wide range of different applications, and the biodegradability is very important when thinking about environmentally-friendly processes and applications. Nevertheless, this paper presents challenges that polymer-based substrates do not present; for example, the porosity or surface roughness of the substrate material, which are crucial parameters when printing conductive lines. However, with polymer-based substrates, one of the crucial parameters is the glass transition temperature (T_g_), due to the use of temperature during the fabrication process [1].

Inkjet-printing enables cost-efficient mass manufacturing of electrodes and other functional materials on large and flexible substrates, such as plastic [2], paper [3], and fabrics [4], with a broad area of applications and huge business potential. First, inkjet-printing enables 3D-printed structures, changing the whole system of producing electronic devices, including the design and manufacturing phases, material selection, and device structure and architecture. Second, printed electronics offer better economics to electronics manufacturing. Traditional electronics are cheap only on the mass production scale, in contrast to digital printing, and especially inkjet-printing, which offers flexible and cheap production for tailored small-volume products (e.g., sheet-to-sheet production). Nevertheless, there are some decisive production parameters that affect conductivity and mechanical properties, such as surface structure of the substrate material, cross-section of the printed layer, and curing parameters like sintering time and temperature [5]. Additionally, the reliability of the printed structures is a crucial matter, such as the variation between electrodes’ width, gap, and uniform layer thickness, for instance. This will influence and limit parameters such as nominal capacitance of the sensor or huge variation in capacitance changes with relative humidity, as well as response time, due to the sensing layer uniformity.

Sintering can be simply explained as a process that welds together small metal particles. It occurs by applying heat to the material below the melting point in such a way that the small metal particles diffuse through the microstructure, coalescing and forming a conductive track, while the organic material is burned off [6]. The most common and simple sintering technique is thermal sintering [7]. This technique usually requires only one oven, with temperatures between 100 and 300 °C for periods of time greater than 30 min. When thinking about roll-to-roll (R2R) mass manufacturing, this is a disadvantage, as it is time consuming. Alternative sintering methods can then be used, significantly reducing the sintering time. Sintering methods such as IR [8], UV [9], flash [10], laser [11], plasma [12], microwave [13], chemical/room temperature [14], or electrical-sintering [15] can be used as an alternative. Specifically, IR-sintering has easy operation, usually only requiring a set of lamps, and is compatible with R2R mass manufacturing. The sintering time is significantly reduced due to localized heating, tailored according to the substrate, such as T_g_, thermal stability, and conductivity.

In a relative humidity capacitive sensor, the dielectric material of a capacitor is allowed to diffuse and absorb water vapour so that it equilibrates with the external environment. The “dry” dielectric constant is significantly lower than the one of water, and thus the electrical capacitance increases as the dielectric absorbs water, hence reflecting the relative humidity (RH) of the surroundings. Usually, continuous and reversible humidity sensing is based on the exposure of thermoset polymers to different relative humidity conditions, followed by reversible dielectric permittivity change [16]. Ideally, thermoset polymer-based capacitive RH sensors detect changes in relative humidity as a change in sensor capacitance with fast response, high linearity, low hysteresis, and excellent long-term stability. Different electrodes configuration can be used for the printed capacitive humidity sensors. The interdigitated electrodes (IDE) configuration is easy to fabricate, but can also detect relative humidity changes in the substrate film and therefore has a slow response time, whereas the well-known parallel-plate configuration detects only relative humidity changes in the thin polymer layer and has a faster response time. Dias et al. [17] described the changes in relative humidity and the influence of the substrate material in capacitive changes for IDE configuration. Additionally, Molina-Lopez et al. [2] showed the proof-of-concept of printed humidity sensors in flexible polymer-based substrates, using capacitive reading. Pleteršek et al. [18] developed a screen-printed humidity sensor on different paper-based substrates, based on capacitive response, with 1 × 1 cm comb type sensor. Recent research has been done in the field of paper electronics where the use of paper as a substrate or active layer for sensor development has been introduced [19,20,21,22,23,24,25]. Han et al. [19] developed a resistive-type humidity sensor fabricated on different cellulose-based papers, with a simple structure, low fabrication cost, and simple read-out circuitry. The sensors were fabricated with single-walled carbon nanotubes, functionalized with carboxylic acid and deposited on the paper substrates. They showed 6% sensitivity in the linear regime and a response time of only 6 s. Gomes et al. [26] studied the effects of humidity on the electrical properties of inkjet-printed films of copper tetrasulfonated phthalocyanine on paper substrates. For instance, it was observed that the resistance values were strongly dependent on the number of printed layers. Courbat et al. [27] used paper as an active layer on IDE configuration inkjet-printed humidity and temperature sensors. They exhibited an exponential response to different humidity steps. Although they achieved better results when the paper was passivated, the sensor showed a very long response time to the humidity changes, independently of the parylene coating.

This paper introduces the use of paper not only as flexible substrate material, but also as active sensing layer, responsive to relative humidity changes. It is an advantage over conventional IDE sensors. It requires no additional steps such as coating or passivation layers, and uses commercial paper. The use of fewer layers introduces less errors and faster fabrication process. Inkjet-printing was used to fabricate the conductive electrodes, using commercial Ag ink. Commercial paper was used as substrate and sensing material. It was exposed to changes in relative humidity from 40 to 100 RH %, at different temperatures from 30 and 85 °C, with an almost linear behaviour, small hysteresis, good response time of less than 5 min, and high reproducibility.

## 2. Materials and Methods

### 2.1. Materials

Lumi silk (90 g/m^2^) from Stora Enso (Helsinki, Finland) was used as substrate material. A commercially available silver (Ag) nanoparticle colloidal ink (ANP 40LT15C) from Advanced Nano Products Co., Ltd. (Sejong, Korea) was used in this work. The metallic nanoparticle-based ink contains around 30 wt % Ag nanoparticles in a polar solvent, with a particle diameter of around 5–10 nm.

### 2.2. Surface Characterization Methods

Optical characterization of the inkjet-printed patterns was made using an optical microscope Olympus BX60 equipped with an Olympus SC30 camera (Olympus Corporation, Shinjuku, Japan) to visually inspect the quality of the printed line. A Veeco optical interferometer equipped with a Wyko NT9100 detector (Veeco, Plainview, Town of Oyster Bay, New York, United States of America) was used to measure the physical parameters of the printed structures (e.g., width and thickness of the printed fingers) and acquire a topographical image on a 5 mm × 5 mm area, with 5× magnification.

### 2.3. Inkjet-Printing and Sintering Conditions

**Figure 1:**
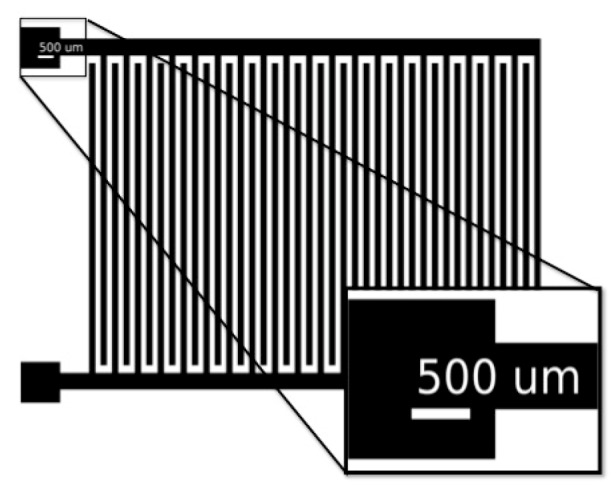
Schematic of the printed interdigitated electrodes (IDE) sensors, with 40 fingers (scale bar represents 500 μm).

Inkjet-printing was carried out using a piezoelectric multi nozzle from Dimatix (Santa Clara, CA, USA, DMP-2831), with 10 pL cartridges. The drop spacing was about 30 µm. While printing, the substrate temperature was set to 60 °C in order to improve the evaporation of the solvent from the ejected droplets. The ink-jetted structures were printed onto the substrates without any pre-treatment. In the IDE configuration, the line width of the fingers structure (40 fingers in total, 20 fingers for each electrode) was set to 200 μm, with 150 μm gap and 900 μm length, occupying a total surface area of 1 cm × 2 cm as in Figure 1.

Infra-red (IR) was used for sintering the printed devices, using an IR-oven Infrared IC heater T-962 Puhui (from Puhui Electric Technology Co., Ltd., Taian, China) with 800 W output power. After the printing of the structures, the substrates were introduced in an IR-oven at 160 °C for 7 min.

### 2.4. Capacitive Humidity Measurements

Changes in capacitance were measured using an HP 4192A LF impedance analyser (5 Hz–13 MHz) at a fixed frequency of 100 kHz every 10 s. The choice of frequency was due to its compatibility with wireless read-out. The devices were chosen and connected to the impedance analyser and were introduced in an SH-221, from ESPEC Corp (Tenjinbashi, Japan). humidity chamber with a wet and dry airflow control, at different humidity steps in the range of 40–100 RH % and temperatures in the range of 30 to 85 °C, for a fixed period of 20 min each. The data was collected and recorded through a PC, using in-built software. A commercial sensor Tiny Tag Plus 2, TGP-4500 (from Gemini Data Loggers Ltd., Chichester, UK) was used to register and control the exact temperature and humidity levels inside the humidity chamber every 10 s in order to verify the chamber accuracy. Both sensors, printed and commercial, were placed in the middle of the chamber in order to reach the equilibrium and interact in the same way with the surrounding environment.

## 3. Results and Discussion

### 3.1. Inkjet-Printing of the Sensor Structure on Paper

Typically, the conventional fabrication process requires a sensing material, either deposited or printed on top of the IDE. In this work, no such layer was introduced, using the paper as substrate and as sensing material. A piezoelectric inkjet-printer was used to print the patterns in an IDE configuration. This configuration was designed as shown in Figure 1. After the inkjet-printing of the IDE structure, the quality of the printed lines was visualized. Fabrication yield was between 60% and 70%, in a sheet-to-sheet production mode.

**Figure 2:**
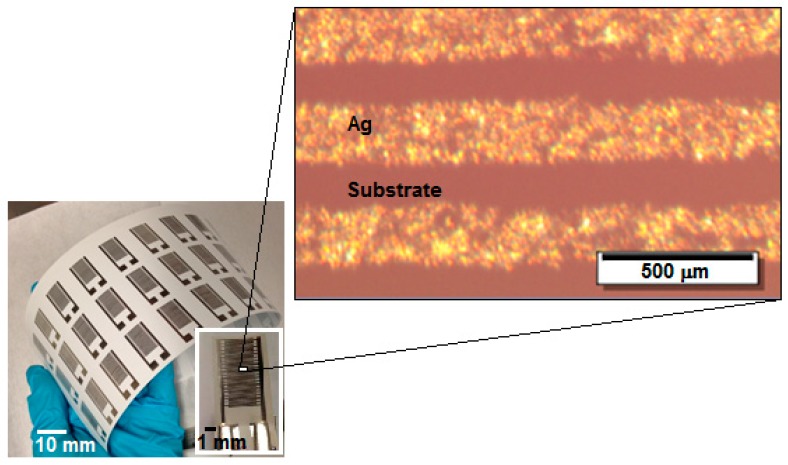
Digital picture of an array of inkjet-printed Ag interdigitated finger electrodes structure and the magnified view of one device and the electrodes (5× magnification).

Optical microscope images were taken of the surface of the IR-sintered lines on the paper substrates, after sintering (see Figure 2).

Physical dimensions of the finger electrodes were measured with the optical profilometer, and the printed structures showed average width of 208.0 ± 15.7 µm, a gap between fingers of 140.0 ± 9.4 µm, and average thickness of 185.4 ± 69.4 nm. These values are slightly discrepant from the aimed dimensions due to the wetting of the ink on the paper surface and the paper porosity. Usually, during the process of depositing ink onto the substrate surface of the paper, a part of the ink immediately soaks the paper fibres and penetrates the paper. This not only promotes the drying process, increasing the evaporation rate of the ink solvent, but also improves adhesion. Simultaneously, it improves high print resolution, avoiding spreading on the substrate surface, improving the edge line roughness definition. From Figure 2, the optical image exhibits a uniform surface, without disruptions, holes, or cracks, and an absence of the coffee-ring effect after printing. The coffee-ring effect can be detrimental to the conductivity of the printed lines, so it is best to be avoided. It has been discussed greatly by other groups [1,8,28], so it will not be thoroughly analysed in this paper. As a quick remark, it does not have a very pronounced effect in porous substrates. Nevertheless, it is important to highlight that it has a more pronounced effect when using thermal sintering methods, or, for instance, with polymer-based substrates. At lower temperatures of around 130–150 °C, commonly used in thermal sintering, there is time for particle movement due to the evaporation of ink solvents. On the other hand, using photonic sintering methods, that effect is almost non-existent. The energy of the photonic radiation is more localized and efficient, meaning that the solvent evaporation and the coalescence of the nanoparticles happen almost at the same rate, not allowing the nanoparticles to move towards the edges of the printed lines. The use of high sintering temperatures—normally above 150 °C and for long periods of time (greater than 30 min)—promotes the yellowing or aging of the fibbers. That effect was studied and discussed in our previous work [29]. In this work, the photonic sintering method used did not promote the yellowing of the paper fibres, as the used temperature and time were not high or long enough, respectively. Another common effect when using thermal sintering is crack formation, or microcracking [8], resulting in disruptive and non-conductive structures. Paper has a low thermal expansion when compared to polymers, and that is the main reason why the crack formation due to substrate shrinkage is almost non-existent [9].

In the author’s previous work [29], a set of three different paper substrates and one polymer substrate—all commercially available—were studied in order to find the most suitable paper for inkjet-printed conductive inks. Hence, Lumi Silk was chosen as the most suitable substrate to use as a substrate for inkjet-printing conductive structures. Other commercially available paper substrates can be used, in combination with different Ag inks. However, for R2R mass manufacturing processes, it is more convenient to choose readily-available materials, so as to make the process faster, of easy-operation, and low-cost.

### 3.2. Capacitive Humidity Measurements

Initial capacitive humidity measurements were made in a humidity chamber at a fixed temperature of 40 °C. The printed sensors were tested under a wide humidity range from 40 to 100 RH %, in 20 min intervals, increasing and decreasing the humidity steps. The humidity levels were set up in this interval due to the physical constraints of the humidity chamber. The selected time period was set up to 20 min in order to allow the diffusion of water vapour molecules to be adsorbed into the substrate and stabilize, reaching the equilibrium with the environment. The output signals of both sensors were normalized to the nominal value of capacitance C_0_. Relative humidity values come from the wet and dry airflow control.

**Figure 3:**
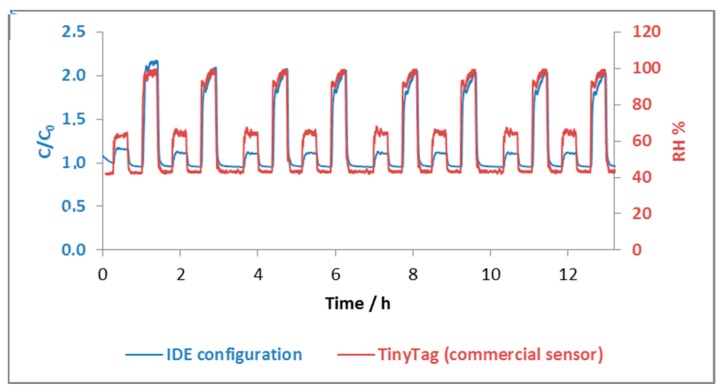
Dynamic response of an IDE configuration printed sensor (in blue) against a commercial sensor, at a fixed frequency of 100 kHz.

Good reproducibility of the printed sensor is shown in Figure 3, over a minimum period of 12 h when exposed to alternate different humidity levels, at room temperature. The dynamic response of the printed sensor against the response of the commercial sensor shows that at high humidity rates (over 60 RH %), the printed sensor response approaches and almost matches the commercial sensor response. Additionally, for lower humidity rates (below 60 RH %), the sensor response does not match the commercial sensor.

This behaviour shows hysteresis of the printed sensor in an up and down cycle. This might be due to saturation of the paper substrate sensing material. The explanation for that is quite simple and it is due to the adsorption and desorption process for an IDE sensor, when exposed to different relative humidity levels. When the relative humidity is low (below 40 RH %), the sensing material will uptake and adsorb the water vapour molecules, usually swelling rapidly. If the relative humidity increases to higher levels (e.g., above 80 RH %), the sensing material will swell more, adding more water vapour molecules adsorbed to the previous ones. After that, if the relative humidity is reduced to the initial level (40 RH %), the sensing material will take more time to desorb and to reach the equilibrium with the surroundings. That usually translates into a slow de-swelling, due to saturation. The transition between the swell and de-swell steps depends on the sensitivity of the material towards the uptake of water vapour molecules and the ability to desorb them and reach equilibrium. In the case of paper substrate, resuming the adsorption process might be relatively fast, whereas desorption and the process to reach equilibrium state will be slower, due to the soaking of the fibres in paper.

**Figure 4:**
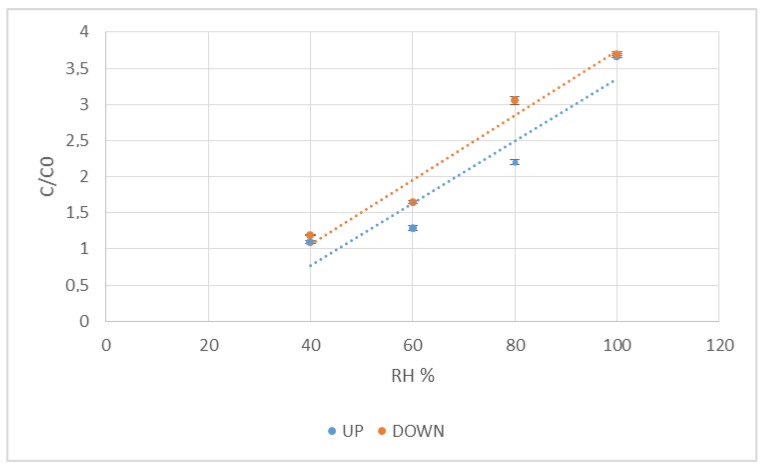
Inkjet-printed sensor linearity, in an up-down cycle in the interval of 40–100 RH %. The UP (in blue) and DOWN (in red) curves relate to the adsorption and desorption cycles, respectively. The data represent means ± SD (*n* = 10).

Another way to represent the UP and DOWN cycles—or the hysteresis of the inkjet-printed sensor—is represented in Figure 4.

These UP and DOWN cycles refer to an increase in relative humidity (UP) and a decrease in relative humidity (DOWN) for the entire measurement cycle. The coefficient of linearity is 0.90 and 0.96, as calculated for the UP and DOWN cycles, respectively. It is then inferred that the inkjet-printed sensors have an almost linear response against humidity, which translates into a small hysteresis loop after long-term operation. This means that the response is very similar in different relative humidity levels. Hysteresis is a typical and significant problem in capacitive sensors. As explained before, it derives from the absorbed water inside the paper structure, increasing the swelling of the fibres, making it slower to desorb and reach the equilibrium, causing a variation in the linearity of the sensor, and thus, hysteresis. It can be improved by reducing the paper thickness, because if the adsorption layer is thinner [30], it will require less time to desorb water vapour molecules and therefore reach the equilibrium state. The sensor can then have an improvement in hysteresis and response time.

**Figure 5:**
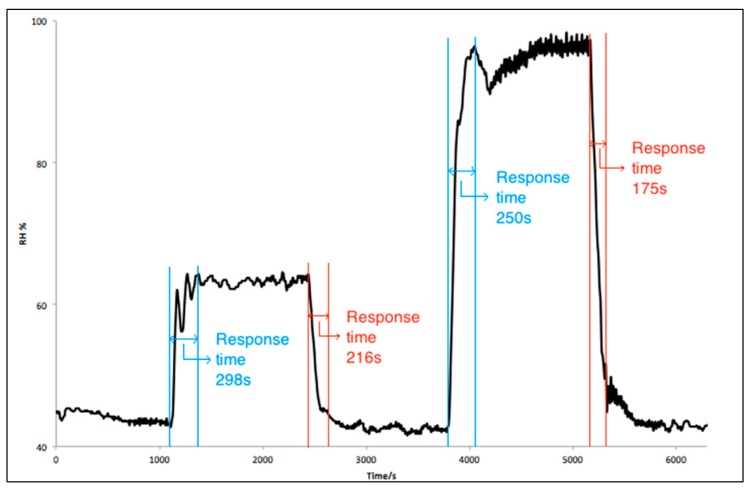
Transient response of the inkjet-printed sensor.

The response time of the IDE inkjet-printed sensors on paper substrate was calculated for humidity steps from 40 to 100 RH %, as represented in Figure 5.

The response time of the IDE printed sensor was about 5 min, whereas the recovery time of the IDE printed sensor was less than 4 min for about 60 RH %. For high humidity levels of 100 RH %, the response time of the IDE printed sensor was about 4 min, whereas the recovery time of the IDE printed sensor was less than 3 min. Although the printed sensors cannot compete with the commercial humidity sensors, with response times between 4 s and 47 s [31,32,33], our printed sensor had a faster response than some printed sensors on flexible substrates reported in the literature [26,27,34], as shown further along in Table 1. Although a response time of 5 min can be considered a long time, if compared with a carbon nanotubes (CNTs)/paper-based resistive sensor [19], it can be used for different applications where there is no need for a fast response or measurement. For instance, in packaging [25] or environmental monitoring [34], these types of sensors can be useful, where the humidity changes are mostly gradual and slow.

The effect of the different temperatures on the relative humidity response and on the repeatability of the sensor performance was also studied with the inkjet-printed sensors, with a minimum of three parallel samples. Temperature is one of the most important parameters that interferes with the response of the inkjet-printed sensors.

**Figure 6:**
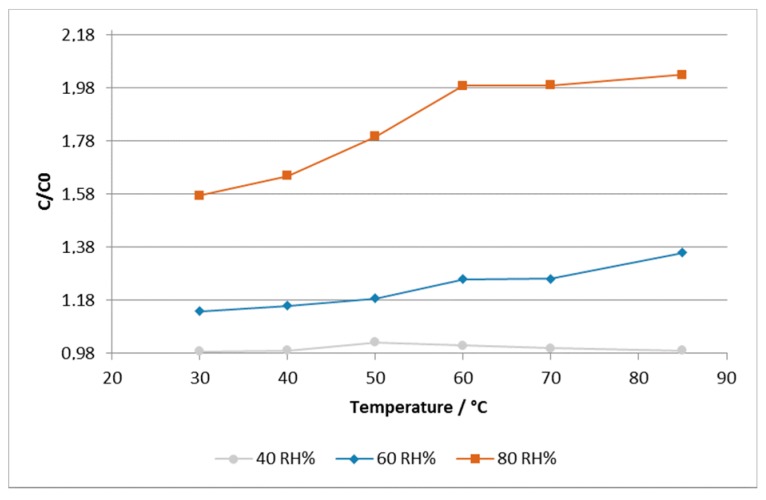
Temperature influence on the capacitance of the inkjet-printed sensor at different humidity rates, for a fixed frequency of 100 kHz.

Figure 6 shows that the sensors are somewhat sensitive to humidity changes between 40 and 80 RH %, when exposed to different temperature ranges within 30–85 °C.

For lower humidity rates (around 40 RH %), the sensors show small sensitivity to temperature changes, whereas above this humidity level, the sensors show higher sensitivity. The temperature effect takes place at 60 °C and remains somewhat the same up to 85 °C, being more pronounced at higher humidity rates (e.g., 60 and 80 RH %). Above 85 °C, the sensors show some instability, probably due to degradation of the substrate and/or the printed layer. The temperature sensitivity of the inkjet-printed sensors can become negligible when applied to low-cost, mass-manufactured environmental sensors, as referred to previously. Nevertheless, the achieved performance was considered acceptable for inkjet-printed moisture sensors on paper substrate when compared with traditional commercial sensor and other printed sensors reported in the literature, as is summarized in Table 1 [19,26,27,34].

Courbat et al. [27] developed an IDE humidity sensor on paper, with similar dimensions to the inkjet-printed sensor described in this work. The main difference is that it uses a sensing material responsive to humidity variations, and the paper acts only as a support. A differential method was used to eliminate the substrate effects when exposed to humidity. Their sensors showed slight hysteresis, with a very long response time as reported. Another issue was that the sensors bent irreversibly when exposed to high levels of humidity. They required paper and electrodes passivation in order to overcome that phenomenon. Compared with our developed sensors, they performed with no mechanical issues, at low and high humidity levels. Between 40 and 100 RH %, our sensors showed an almost linear behaviour and response time of less than 5 min, which is faster than most of the sensors reported in the literature [26,27,34].

To improve the sensor sensibility and simultaneously reduce the oxidation of Ag, a well-known phenomenon due to exposure to humidity, electrodeposition of a thin metal layer can be performed. This will be addressed in future works as a way to reduce the influence of humidity and temperature on the sensor response. The electrodeposition of a thin film of nickel and copper as a way to passivate the inkjet-printed lines has been described in previous works [2,35]. This passivation plays an important role in improving the sensor sensitivity, as well as for the integration of temperature and gas devices. The use of other conductive inks which are more inert to environmental conditions (e.g., carbon [36]) can also be addressed in future works, as a way to improve the sensor performance.

## 4. Conclusions

All-inkjet-printed capacitive-type humidity sensors on a paper substrate which is sensitive to moisture were demonstrated herein. Inkjet-printing was shown to be a convenient, easy, fast, and cheap way to produce IDE configuration relative humidity sensors on flexible substrates such as paper with a good printing quality, resolution, and high fabrication yield. Inkjet-printing has the advantage, as a sheet-to-sheet (S2S) production technique, to be up-scalable to roll-to-roll (R2R) mass manufacturing, thus reducing drastically the productions costs. The realized inkjet-printed paper sensors showed to be comparable to commercial sensors [31,32,33] in terms of performance against humidity changes. They also showed a faster response time than the inkjet-printed humidity sensor on paper realized by Courbat et al. [27], for example, as well as others reported in the literature [26,34]. Despite the somewhat slower response time, the all-inkjet-printed sensors can be used in applications that do not require fast response time (e.g., environmental monitoring), integrating them in a label or tag [37,38,39]. Another application could be addressed with fully-printed transistor devices, being indicated in with a display (OLED) [40,41]. Most importantly, the inkjet-printed sensors on flexible paper substrates show several advantages, such as being environmentally-friendly, recyclable, low-cost, foldable, and disposable, and can be easily integrated in different applications.

## Figures and Tables

**Table 1:** Summary of the inkjet-printed humidity sensor performance compared to the sensor performance of commercial and literature sensors. CNTs: carbon nanotubes; CuTsPc: copper tetrasulfonated phthalocyanine.

Parameter	Si-Based Commercial Sensor	Paper-Based Inkjet-Printed Sensor	Polymer-Based Inkjet-Printed Sensor [34]	Inkjet-Printed Humidity Sensor on Paper [27]	CNTs/Paper-Based Resistive Sensor [19]	CuTsPc/Paper-Based Resistive Sensor [26]
Nominal capacitance (pF) @ room temperature	300 (55 RH %)	10 (55 RH %)	2 (40 RH %)	23	n.d.	n.d.
Sensitivity (pF /RH %)	0.6 pF	~ 2 pF	4.5 fF	n.d.	6% (linear regime)	n.d.
Response time	Fast (seconds)	Slow (4–5 min)	6 min	Very long	6 s	8 min
